# Supplementary material for: Techno-Functional and Sensory Characterization of Commercial Plant Protein Powders
Source: Foods. 2023 Jul 24;12(14):2805. doi: 10.3390/foods12142805 (PMC10379337; doi:10.3390/foods12142805)
Supplement: Supplementary file 1 [file foods-12-02805-s001.zip › foods-2508831-supplementary.pdf]

Techno-functional and sensory characterization of commercial plant protein powders

Kadi Jakobson, Aleksei Kaleda, Karl Adra, Mari-Liis Tammik, Helen Vaikma, Tiina Kriščiunaite and Raivo Vilu

Supplementary information

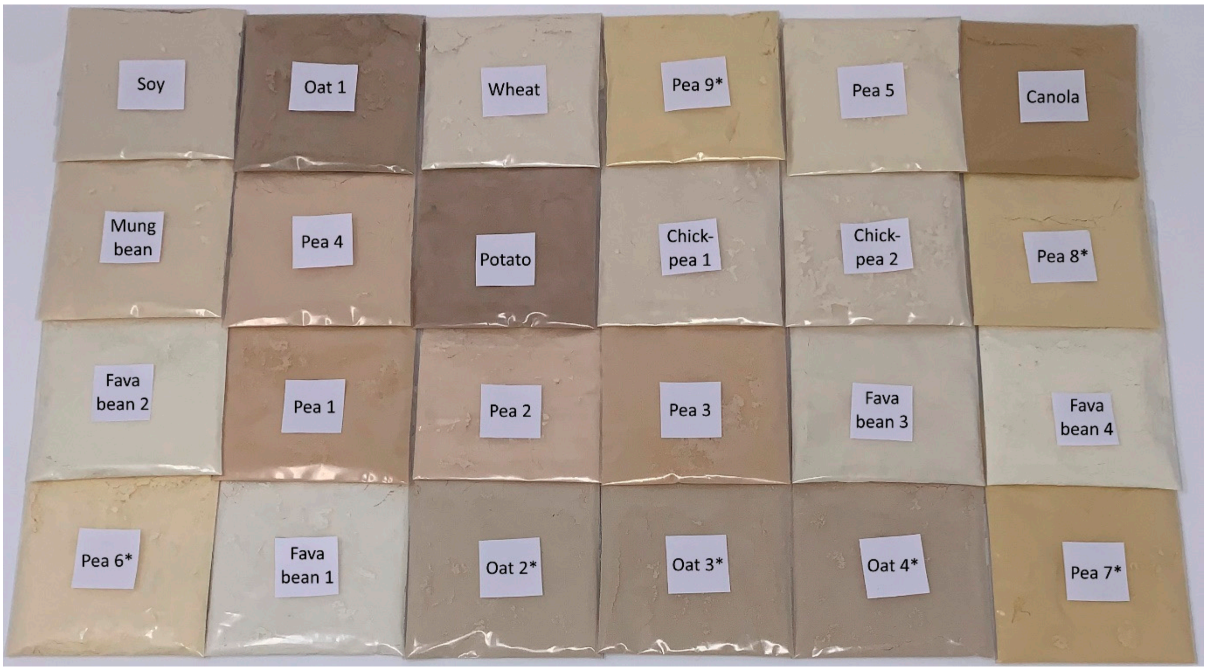

Figure S1. Photo of commercial protein powders prepared for color measurement.

Table S1. Nutritional composition according to manufacturers' specifications and native pH.

Sample	Protein, % dwb	Fat, % dwb	Carbohydrates, % dwb	Ash, % dwb	Native pH
Canola	90	-	-	5	6.13
Chickpea 1	89	0	1	4	6.95
Chickpea 2	89	0	2	3	6.95
Fava bean 1	60	4	25	-	6.54
Fava bean 2	60	4	-	-	6.70
Fava bean 3	88	5	1	6	6.45
Fava bean 4	60	3	18	-	6.72
Mung bean	85	4	1	3	7.07
Oat 1	59	9	27	4	6.01
Oat 2*	56	13	21	3	6.55
Oat 3*	56	13	21	3	6.55
Oat 4*	56	13	21	3	6.61
Pea 1	85	-	-	5	7.35
Pea 2	85	9	0	6	7.39
Pea 3	84	9	0	5	7.46
Pea 4	80	5	2	9	7.10
Pea 5	90	-	-	4	7.56
Pea 6*	80	9	4	5	7.02
Pea 7*	80	9	4	5	6.60
Pea 8*	80	9	4	5	7.10
Pea 9*	80	9	4	5	7.11
Potato	90	0	0	5	7.32
Soy	90	1	-	6	7.74
Wheat	82	5	8	1	6.26

Table S2. CIELAB color. Mean (SD, n = 3).

Sample	L*	a*	b*
Canola	73.86 (0.11)	4.01 (0.04)	24.31 (0.08)
Chickpea 1	93.64 (0.17)	-0.37 (0.04)	18.45 (0.04)
Chickpea 2	94.49 (0.01)	-0.56 (0.11)	10.57 (0.05)
Fava bean 1	85.04 (0.07)	1.80 (0.08)	14.05 (0.12)
Fava bean 2	82.97 (0.22)	1.91 (0.59)	14.86 (0.06)
Fava bean 3	85.49 (0.06)	1.95 (0.09)	26.45 (0.06)
Fava bean 4	82.94 (0.13)	1.85 (0.14)	14.44 (0.10)
Mung bean	74.38 (0.11)	2.88 (0.10)	16.27 (0.10)
Oat 1	83.20 (0.29)	4.15 (0.09)	18.95 (0.06)
Oat 2*	94.67 (0.13)	-0.69 (0.08)	12.73 (0.04)
Oat 3*	83.10 (0.13)	4.39 (0.09)	18.57 (0.11)
Oat 4*	87.84 (0.17)	3.44 (0.03)	15.38 (0.12)
Pea 1	88.90 (0.26)	1.04 (0.10)	17.85 (0.06)
Pea 2	85.16 (0.23)	3.43 (0.04)	17.31 (0.06)
Pea 3	73.46 (0.09)	4.03 (0.02)	15.73 (0.09)
Pea 4	89.37 (0.03)	-0.06 (0.05)	15.21 (0.09)
Pea 5	88.83 (0.13)	-0.13 (0.13)	14.46 (0.09)
Pea 6*	91.01 (0.28)	-0.15 (0.17)	12.81 (0.28)
Pea 7*	94.63 (0.18)	-0.83 (0.15)	12.42 (0.09)
Pea 8*	89.56 (0.07)	0.08 (0.03)	14.80 (0.03)
Pea 9*	87.05 (0.08)	0.96 (0.04)	24.78 (0.12)
Potato	90.52 (0.28)	-0.14 (0.10)	13.06 (0.17)
Soy	87.87 (0.22)	0.36 (0.07)	25.40 (0.13)
Wheat	90.89 (0.34)	-0.21 (0.03)	15.70 (0.05)

Table S3. Solubility. Mean (SD, n = 3).

Sample	WSI [%]	Solubility, native pH [%]	Solubility, pH 4.5 [%]
Canola	88.7 (0.1)	13.9 (0.7)	10.7 (0.4)
Chickpea 1	16.4 (0.1)	26.7 (0.4)	4.1 (0.2)
Chickpea 2	26.2 (1.2)	31.7 (0.4)	19.7 (0.3)
Fava bean 1	59.3 (0.4)	49.7 (0.8)	20.4 (0.7)
Fava bean 2	42.5 (0.4)	43.4 (0.3)	18.7 (0.1)
Fava bean 3	8.7 (0.3)	5.5 (0.3)	3.2 (0.1)
Fava bean 4	47.3 (0.5)	52.1 (0.1)	27.3 (0.4)
Mung bean	15.3 (1.1)	6.4 (2.8)	7.9 (0.3)
Oat 1	3.8 (0.1)	4.9 (0.1)	6.2 (0.3)
Oat 2*	9.7 (0.0)	19.5 (0.2)	18.8 (0.5)
Oat 3*	8.8 (0.1)	16.5 (0.1)	16.9 (0.2)
Oat 4*	9.8 (0.1)	17.3 (0.4)	17.7 (0.4)
Pea 1	36.7 (0.0)	45.8 (0.0)	24.4 (0.0)
Pea 2	18.2 (0.2)	54.4 (0.4)	4.2 (0.4)
Pea 3	17.2 (0.1)	52.9 (0.5)	4.3 (0.3)
Pea 4	11.3 (0.3)	33.6 (0.3)	2.9 (0.4)
Pea 5	42.1 (1.2)	92.7 (0.3)	16.5 (0.9)
Pea 6*	42.6 (0.3)	13.9 (0.3)	5.8 (0.2)
Pea 7*	9.5 (0.1)	12.1 (0.4)	7.7 (0.1)
Pea 8*	16.3 (0.1)	37.5 (0.4)	35.1 (0.3)
Pea 9*	13.9 (0.1)	36.1 (0.3)	7.2 (0.5)
Potato	94.3 (0.0)	0.0 (0.0)	0.0 (0.0)
Soy	24.3 (3.1)	93.6 (1.7)	0.1 (0.5)
Wheat	8.6 (0.5)	12.8 (0.5)	39.4 (2.8)

Table S4. Water- and oil-holding capacities. Mean (SD, *n* = 3).

Sample	WHC [g(H_2O) g^{-1}]	OHC [g(oil) g^{-1}]
Canola	-	2.77 (0.05)
Chickpea 1	2.56 (0.03)	1.45 (0.00)
Chickpea 2	1.20 (0.08)	1.28 (0.06)
Fava bean 1	0.62 (0.00)	1.09 (0.00)
Fava bean 2	1.06 (0.01)	0.90 (0.02)
Fava bean 3	1.85 (0.04)	1.14 (0.05)
Fava bean 4	0.92 (0.01)	0.83 (0.01)
Mung bean	2.31 (0.12)	1.66 (0.01)
Oat 1	1.71 (0.08)	1.13 (0.02)
Oat 2*	1.49 (0.01)	0.87 (0.01)
Oat 3*	1.47 (0.04)	0.96 (0.01)
Oat 4*	1.47 (0.00)	0.87 (0.00)
Pea 1	1.17 (0.01)	1.14 (0.01)
Pea 2	3.12 (0.05)	0.80 (0.01)
Pea 3	3.22 (0.02)	1.28 (0.00)
Pea 4	2.80 (0.03)	1.22 (0.01)
Pea 5	3.52 (0.05)	1.61 (0.32)
Pea 6*	0.85 (0.01)	0.85 (0.01)
Pea 7*	1.63 (0.07)	0.79 (0.01)
Pea 8*	2.19 (0.01)	1.01 (0.01)
Pea 9*	2.27 (0.02)	1.07 (0.01)
Potato	-	2.09 (0.10)
Soy	6.27 (0.02)	1.64 (0.09)
Wheat	1.47 (0.00)	0.87 (0.01)

Table S5. Foaming and emulsification properties. Mean (SD, n = 2).

Sample	FC [%]	FS [%]	EA [%]	ES [%]
Canola	67.1 (1.7)	58.1 (5.7)	51.6 (2.3)	67.7 (3.3)
Chickpea 1	62.8 (0.0)	51.4 (2.0)	47.8 (0.0)	50.5 (0.8)
Chickpea 2	84.1 (5.2)	39.5 (1.3)	46.8 (3.0)	51.1 (0.0)
Fava bean 1	44.8 (2.2)	69.9 (4.6)	51.7 (0.8)	48.1 (1.1)
Fava bean 2	21.5 (0.0)	55.9 (3.3)	51.1 (0.0)	53.1 (1.2)
Fava bean 3	41.9 (3.3)	66.6 (2.0)	51.6 (0.7)	52.2 (0.0)
Fava bean 4	24.4 (1.6)	53.6 (0.4)	51.1 (1.5)	51.6 (0.8)
Mung bean	32.6 (0.0)	82.2 (0.6)	18.8 (2.9)	45.4 (6.5)
Oat 1	14.3 (0.0)	0.0 (0.0)	0.0 (0.0)	4.1 (0.1)
Oat 2*	10.7 (1.7)	7.1 (10.1)	22.9 (2.9)	41.2 (2.3)
Oat 3*	9.4 (0.2)	0.0 (0.0)	12.7 (6.1)	35.4 (1.9)
Oat 4*	18.6 (3.3)	0.0 (0.0)	17.9 (4.2)	44.3 (2.1)
Pea 1	44.8 (0.9)	53.6 (7.7)	48.9 (1.5)	52.6 (2.2)
Pea 2	44.2 (0.0)	43.6 (2.7)	51.0 (1.5)	50.5 (0.8)
Pea 3	34.9 (6.6)	68.4 (13.2)	49.4 (0.8)	52.2 (0.0)
Pea 4	47.7 (1.6)	49.0 (4.0)	51.0 (1.5)	50.5 (2.2)
Pea 5	41.8 (2.9)	53.4 (3.3)	52.5 (0.4)	50.0 (0.8)
Pea 6*	21.8 (0.8)	40.7 (5.2)	49.5 (0.7)	51.4 (6.3)
Pea 7*	23.3 (3.3)	31.0 (3.4)	50.5 (0.8)	50.5 (0.8)
Pea 8*	22.7 (0.0)	57.1 (0.0)	51.6 (2.3)	50.0 (1.5)
Pea 9*	26.7 (1.6)	54.8 (2.1)	48.9 (1.5)	53.1 (1.5)
Potato	95.3 (0.0)	66.3 (1.4)	49.5 (0.8)	51.6 (0.7)
Soy	55.3 (1.1)	70.2 (11.9)	51.1 (1.5)	52.6 (2.2)
Wheat	97.7 (3.3)	54.1 (1.1)	51.6 (0.7)	51.6 (2.3)

Table S6. Sensory properties. Mean (SD, n = 9).

Sample	O. Overall odor intensity	O. Raw material odor	O. Off-odor intensity	T. Overall taste intensity	T. Raw material taste	T. Bitterness	T. Astringency	T. Off-taste intensity	X. Particle size	X. Amount of particles
Canola	8.6 (0.5)	7.7 (1.0)	1.0 (1.0)	7.4 (0.9)	6.4 (0.9)	2.1 (0.9)	5.0 (0.9)	1.0 (1.0)	0.7 (0.9)	0.7 (0.9)
Chickpea 1	8.5 (0.5)	8.3 (0.8)	0.5 (0.8)	6.8 (1.0)	6.0 (0.9)	3.7 (0.8)	3.7 (1.0)	0.0 (0.0)	1.5 (0.5)	4.0 (0.9)
Chickpea 2	7.7 (0.5)	7.2 (1.0)	0.5 (0.8)	7.0 (0.9)	6.8 (1.0)	4.8 (1.0)	5.7 (1.0)	1.5 (0.5)	1.8 (1.0)	5.0 (0.9)
Fava bean 1	8.7 (0.8)	8.7 (0.8)	0.0 (0.0)	8.0 (0.9)	8.0 (0.9)	5.8 (1.0)	5.2 (1.0)	0.0 (0.0)	1.3 (0.5)	2.0 (0.6)
Fava bean 2	7.7 (0.5)	7.3 (0.8)	0.7 (0.8)	7.2 (0.8)	6.7 (0.8)	5.2 (1.0)	5.7 (1.0)	0.0 (0.0)	1.5 (0.8)	4.7 (1.0)
Fava bean 3	8.0 (0.6)	7.5 (0.5)	0.0 (0.0)	6.2 (1.0)	5.8 (1.0)	4.2 (0.8)	3.7 (0.5)	0.0 (0.0)	2.0 (0.6)	5.0 (0.9)
Fava bean 4	6.3 (1.0)	6.0 (0.9)	0.0 (0.0)	6.2 (1.0)	5.7 (0.8)	5.2 (1.0)	4.5 (1.0)	0.0 (0.0)	1.2 (0.4)	4.0 (0.9)
Mung bean	8.7 (0.5)	8.2 (1.0)	3.8 (1.0)	6.7 (0.8)	5.8 (0.8)	3.3 (1.0)	4.0 (0.9)	3.7 (0.8)	2.8 (1.0)	6.3 (0.5)
Oat 1	6.9 (0.9)	5.3 (1.0)	2.0 (1.0)	5.4 (0.5)	4.9 (0.9)	2.9 (0.9)	2.9 (0.9)	0.0 (0.0)	1.9 (0.9)	6.7 (0.5)
Oat 2*	6.8 (0.4)	6.7 (0.8)	0.5 (0.5)	6.0 (0.0)	5.8 (0.4)	2.2 (1.0)	2.7 (1.0)	0.0 (0.0)	1.7 (0.8)	4.3 (0.8)
Oat 3*	7.0 (0.9)	7.0 (0.9)	0.0 (0.0)	5.7 (0.8)	5.7 (0.8)	1.5 (0.8)	2.3 (0.8)	0.0 (0.0)	1.7 (0.8)	4.2 (1.0)
Oat 4*	6.8 (0.4)	6.8 (1.0)	0.0 (0.0)	5.2 (0.8)	5.0 (0.6)	2.0 (0.9)	2.7 (0.8)	0.0 (0.0)	2.0 (0.9)	4.3 (1.0)
Pea 1	6.5 (0.5)	5.7 (0.8)	2.2 (0.8)	6.3 (0.8)	6.0 (0.9)	4.8 (1.0)	5.2 (1.0)	0.0 (0.0)	2.8 (1.0)	5.2 (0.8)
Pea 2	6.3 (0.5)	6.3 (0.5)	0.0 (0.0)	6.0 (0.9)	6.0 (0.9)	4.8 (1.0)	5.0 (0.6)	0.0 (0.0)	1.7 (0.5)	4.3 (0.8)
Pea 3	7.5 (0.8)	7.5 (0.8)	0.0 (0.0)	6.3 (0.8)	6.2 (0.8)	4.7 (1.0)	4.5 (0.8)	0.0 (0.0)	2.0 (0.0)	4.7 (0.5)
Pea 4	8.0 (0.0)	6.2 (0.4)	2.2 (1.0)	6.0 (0.6)	5.2 (0.8)	3.3 (0.8)	4.5 (0.5)	1.2 (1.0)	1.5 (0.5)	4.2 (1.0)
Pea 5	7.8 (1.0)	6.8 (1.0)	0.0 (0.0)	6.2 (0.8)	5.1 (0.9)	5.2 (0.8)	5.3 (1.0)	0.0 (0.0)	0.9 (0.9)	1.0 (0.9)
Pea 6*	7.5 (0.5)	6.0 (0.9)	1.8 (0.8)	5.8 (0.8)	5.2 (1.0)	2.2 (1.0)	2.8 (1.0)	0.0 (0.0)	2.3 (1.0)	4.8 (1.0)
Pea 7*	6.8 (1.0)	5.8 (1.0)	0.7 (1.0)	5.5 (1.0)	4.8 (0.8)	2.0 (0.9)	2.2 (0.8)	0.0 (0.0)	3.0 (0.9)	4.8 (1.0)
Pea 8*	8.3 (0.5)	7.9 (0.9)	1.9 (0.9)	5.1 (0.9)	5.0 (1.0)	2.7 (1.0)	3.6 (0.5)	0.6 (1.0)	1.1 (0.4)	3.3 (1.0)
Pea 9*	8.6 (0.5)	7.8 (0.8)	3.7 (0.9)	6.7 (0.7)	6.3 (1.0)	3.6 (1.0)	4.2 (1.0)	0.7 (1.0)	1.8 (0.8)	3.2 (1.0)
Potato	8.5 (0.5)	6.7 (0.8)	1.8 (1.0)	6.0 (0.6)	5.5 (0.8)	2.2 (1.0)	2.8 (1.0)	0.7 (1.0)	0.5 (0.5)	0.8 (1.0)
Soy	7.0 (1.0)	6.0 (1.0)	0.4 (0.8)	5.0 (1.0)	4.9 (0.9)	4.0 (1.0)	3.7 (1.0)	0.0 (0.0)	0.3 (0.5)	0.3 (0.5)
Wheat	8.0 (1.0)	7.2 (1.0)	1.7 (0.9)	5.2 (1.0)	3.4 (0.9)	2.7 (0.9)	4.2 (1.0)	0.3 (0.7)	2.9 (0.9)	2.4 (0.7)
